# Antibacterial PET-G/ZnO Composites: A New Approach to Relaxation Splint Materials in the Treatment of Bruxism

**DOI:** 10.3390/ma19143033

**Published:** 2026-07-14

**Authors:** Sandra Wąsik, Rafał Bielas, Roksana Wygoda, Mateusz Wojciechowski, Magdalena Tarnacka, Krzysztof Aniołek, Anna Mertas, Maciej Zubko, Karsten Manterys, Izabela Barszczewska-Rybarek, Stefan Baron, Małgorzata Karolus

**Affiliations:** 1 International Environmental Doctoral School, Faculty of Natural Sciences, University of Silesia, Bedzinska 60, 41-200 Sosnowiec, Poland; sandra.wasik@us.edu.pl; 2 Institute of Materials Engineering, University of Silesia in Katowice, 75 Pulku Piechoty 1A, 41-500 Chorzow, Poland; roksana.wygoda@us.edu.pl (R.W.); krzysztof.aniolek@us.edu.pl (K.A.); maciej.zubko@us.edu.pl (M.Z.); karsten.manterys@us.edu.pl (K.M.); 3 Department of Pharmacognosy and Phytochemistry, Faculty of Pharmaceutical Sciences in Sosnowiec, Medical University of Silesia in Katowice, Jagiellonska 4, 41-200 Sosnowiec, Poland; rafal.bielas@sum.edu.pl; 4 Department of Temporomandibular Disorders, Medical University of Silesia in Katowice, Traugutta Sq. 2, 41-800 Zabrze, Polandsbaron@sum.edu.pl (S.B.); 5 Institute of Physics, Faculty of Science and Technology, University of Silesia in Katowice, 75 Pulku Piechoty 1A, 41-500 Chorzow, Poland; magdalena.tarnacka@us.edu.pl; 6 Department of Microbiology and Immunology, Faculty of Medical Sciences in Zabrze, Medical University of Silesia, Jordana 19 Street, 41-808 Zabrze, Poland; amertas@sum.edu.pl; 7 Department of Physical Chemistry and Technology of Polymers, Faculty of Chemistry, Silesian University of Technology, 44-100 Gliwice, Poland; izabela.barszczewska-rybarek@polsl.pl

**Keywords:** zinc oxide, copolymer of ethylene terephthalate and 1,4-cyclohexanedimethanol terephthalate (PET-G), relaxation splints

## Abstract

**Highlights:**

**What are the main findings?**

- A novel method for the fabrication of poly(ethylene terephthalate-co-1,4-cyclohexanedimethylene terephthalate) (PET-G) composites incorporating zinc oxide (ZnO) has been developed.
- The incorporation of zinc oxide results in only minor changes to the mechanical properties of the material.
- The addition of zinc oxide significantly enhances the antibacterial performance of the PET-G composite.

**What are the implications of the main findings?**

- The combined melt-processing and solvent-based fabrication approach enables the production of materials with mechanical properties comparable to those of conventional PET-G materials.
- The antibacterial activity of the material can be tailored through appropriate adjustment of the zinc oxide concentration.
- PET-G composites modified with zinc oxide represent a promising material for dental applications, particularly for the fabrication of occlusal splints and relaxation splints.

**Abstract:**

Bruxism-induced wear and microbial colonization of oral splints remain significant challenges in restorative dentistry. In this study, we developed a series of antibacterial nanocomposites based on a poly(ethylene terephthalate-co-1,4-cyclohexanedimethylene terephthalate) (PET-G) matrix incorporated with zinc oxide (ZnO) particles (3, 7, and 10 wt%), designed for the next generation of oral appliances. The composites were fabricated via a solvent-casting method followed by thermal processing. Differential scanning calorimetry (DSC) revealed that the amorphous nature and glass transition temperature (Tg ≈ 80 °C) of the PET-G matrix remained stable, ensuring excellent processability. While a minor decrease in Shore D hardness and flexural strength (up to 15%) was observed due to filler-induced structural discontinuity, the mechanical integrity remained within clinically acceptable limits. Crucially, the PET-G/ZnO composites exhibited potent antimicrobial activity; the 7 and 10 wt% loadings achieved a 100% reduction in *Streptococcus oralis* viability and significant inhibition of *Candida albicans*. These findings demonstrate that ZnO integration provides a dual-functional advantage—maintaining the thermoplastic versatility of PET-G while introducing critical bioactive properties, positioning these materials as a superior alternative for effective long-term bruxism therapy.

## 1. Introduction

Bruxism, defined as a parafunctional disorder, is characterized by pathological, subconscious jaw muscle activity, specifically clenching and teeth grinding, which is considered to have a significant psychosomatic component [1,2]. Although the etiology of bruxism is not precisely defined, many studies have concluded that it has multiple causes, which may overlap and compound one another. The most common causes are morphological, psychosocial, genetic, and other environmental factors [3,4,5,6]. Pathological tooth wear can lead to numerous health complications, including muscle pain, headaches, occlusal trauma, sleep disturbances, and, in some severe cases, bruxism can even cause difficulties with speech or swallowing [7]. Despite growing interest, the epidemiology of this parafunction remains a subject of ongoing discussion and is regularly updated [8,9,10]. Internationally, bruxism is defined as a behaviour or motor activity rather than a pathological condition, assuming the absence of associated diseases [10,11,12,13]. Effective bruxism treatment requires various therapeutic methods, ranging from behavioural therapy and physiotherapy to pharmacotherapy and the use of sleep appliances [10]. One of the most effective methods for limiting the destructive effects of nocturnal bruxism is the use of relaxation splints. Splint therapy involves various types of removable intraoral appliances designed to eliminate occlusal interferences, provide orthopaedic stability, reduce tension, and decrease muscle activity, thereby preventing further dental degradation [10,11,12,14].

Relaxation splints are primarily categorized into soft and hard variants, depending on their material properties and clinical application [13,15,16]. Soft splints are readily available, self-fitted appliances made from a thermoplastic material—ethylene-vinyl acetate copolymer (EVA). Currently available products include Ozdenta^®^, OPRO Night Guard^®^, Bioplast^®^, and Dental Guard^®^. While these types of appliances are often used as mouthguards, they are not recommended for temporomandibular joint disorder (TMD) therapy because their lack of rigidity prevents the achievement of stable occlusion, which is essential for successful treatment [17]. In contrast, hard relaxation splints are most commonly fabricated in a laboratory by a dental technician based on an in-office impression and bite registration of the patient’s dentition. They are typically made of poly(methyl methacrylate) (PMMA) or a copolymer of ethylene terephthalate and 1,4-cyclohexanedimethanol terephthalate (PET-G) [13,15,18,19]. In recent years, other materials with comparable properties [20,21], such as polycarbonate and polyetheretherketone (PEEK), have also emerged as viable alternatives for relaxation splints, offering equally good clinical performance [18,21].

To further enhance clinical outcomes, there is a growing focus on developing composite materials enriched with innovative additives to overcome the mechanical limitations of traditional splint materials [22]. This builds on decades of dental research aimed at improving the properties of materials used for fillings, restorations, and the treatment of various oral conditions [23,24,25]. Among the most widely utilized fillers in dental materials are silver nanoparticles, which enhance antibacterial properties and actively prevent biofilm formation [26,27,28,29]. These nanoparticles are increasingly applied to titanium implants [27,28,29], dentures, dental inserts [30], and for coating dental wires [31,32]. Similarly, silica-containing dental resins have been developed to improve the mechanical properties of fabricated prostheses and dentures [33,34,35]. Other notable reinforcements used in dental composites include zirconia [36,37] and calcium phosphates [38,39,40]. However, one of the key additives that has gained widespread scientific interest is zinc oxide [41,42,43]. Due to its unique antiseptic properties [44,45,46,47,48,49,50,51] and relatively low cost, it has become the subject of extensive scientific research. Zinc oxide has already been used in dentures [52,53] and has proven useful in 3D-printed dental materials [54,55,56,57]. It has been proven not only to influence the mechanical properties [54,58] but also to improve the colour stability of fabricated devices [59].

In contrast to the majority of contemporary studies on dental polymers, which primarily focus on the modification of acrylic resins, prosthetic composites, and materials used for dental tissue reconstruction, the present study investigates PET-G as a material intended for the fabrication of occlusal splints used in the management of bruxism, where maintaining mechanical stability while simultaneously limiting bacterial colonization during long-term intraoral use is of critical importance. Furthermore, most available studies evaluate the effects of functional additives in materials designed for prosthetic restorations and dental rehabilitation, whereas the modification of materials dedicated to bruxism therapy remains a relatively underexplored research area [60,61,62,63,64]. While numerous studies on nanomodified dental polymers have investigated additive concentrations below 5 wt.% due to concerns regarding particle agglomeration and the potential deterioration of material properties [52,53,54], the present work evaluates the effects of higher ZnO loadings, enabling the determination of an effective modification range for PET-G and providing valuable insight into its functional optimization.

Despite their widespread use, currently available relaxation splint materials do not possess sufficient antiseptic properties [19], often acting as reservoirs for oral pathogens that can lead to long-term pathological changes [65,66,67]. Hence, to fill this gap and address the problem of bacterial colonization on splint surfaces, we modified standard PET-G by incorporating zinc oxide (ZnO) at concentrations of 3%, 7%, and 10%. The resulting composites were subsequently subjected to detailed thermal, mechanical, and biological analyses and compared with the base material. These investigations revealed that the new materials not only maintain optimized thermal and mechanical stability but, most importantly, exhibit potent antibacterial activity. These findings mark a significant advancement in dental materials science, offering a high-performance antimicrobial solution specifically engineered to overcome the limitations of traditional bruxism therapy.

## 2. Materials and Methods

### 2.1. Materials

The material used in the study was a commercially available copolymer of ethylene terephthalate and 1,4-cyclohexanedimethanol terephthalate (PET-G) in granular form, obtained from Spectrum Filamets (Pęcice, Poland). The chemical composition of the PET-G copolymer was evaluated via ^1^H NMR analysis, revealing that the material consists of 44% terephthalic acid, 30% ethylene glycol, and 26% 1,4-cyclohexanedimethanol (Figure S1).

These granules served as the base material for the preparation of the composite, which was modified using zinc oxide (ZnO, 98% purity, procured from Warchem (Warsaw, Poland)). The morphology and particle size of the ZnO powder were characterized using SEM (Figure S2) and TEM (Figure S3), revealing a nanocrystalline, faceted morphology (polygonal, plate-like, and short-prismatic grains) with a mean particle size of 157.3 (93.2) nm. Analytical grade dichloromethane (DCM) was purchased from Chempur (Piekary Śląskie, Poland), while phosphate-buffered saline (PBS) was acquired from Sigma Aldrich (St. Louis, MO, USA).

### 2.2. Methods

#### 2.2.1. Preparation of Composite Materials

In each run, 10 g of PET-G polymer granules were dissolved in 100 mL of dichloromethane (DCM). The mixture was stirred until the polymer was completely dissolved, resulting in a clear, viscous solution. Subsequently, powdered zinc oxide was gradually incorporated into the solution in three separate batches to achieve final concentrations of 3%, 7%, and 10% (wt.%). Each mixture was stirred until a homogeneous dispersion was obtained. The resulting suspension was cast and left to dry overnight to evaporate the solvent, forming thin, solid composite films. These films were then heated on a hot plate to 260 °C. Upon reaching a molten state, the material was placed into metal and polytetrafluoroethylene (PTFE) molds to ensure high repeatability and precise specimen geometry. Subsequently, the specimens were dried in a vacuum oven at 40 °C.

#### 2.2.2. Microstructure Analysis with Scanning Electron Microscopy (SEM)

For microstructural imaging using scanning electron microscopy (SEM), the samples were subjected to a two-step gold sputter-coating procedure using a JEOL Smart Coater (JEOL Ltd., Akishima, Tokyo, Japan) system. The coating process was applied to reduce electrostatic charge accumulation on the sample surfaces during SEM observations. In the first stage, all samples were sputter-coated with gold for 3 min. Subsequently, the coated samples were mounted onto aluminum stubs using conductive carbon tape and coated again with a gold layer for an additional 3 min. Microstructural observations were carried out using a JEOL JSM-6480 scanning electron microscope. The images were acquired in secondary electron imaging mode (SEI). To minimize excessive surface charging effects, an accelerating voltage of 10 kV was applied during imaging.

#### 2.2.3. Transmission Electron Microscopy (TEM)

Microstructure analysis was carried out using a JEOL JEM-3010 high-resolution transmission electron microscope (TEM, JEOL Ltd., Tokyo, Japan) operated at 300 kV, equipped with a Gatan 2k × 2k Orius™ 833 SC200D CCD camera (Gatan Inc., Pleasanton, CA, USA). The studied micelles were suspended in isopropanol and deposited onto a Cu grid coated with an amorphous carbon film, standardized for TEM observation. Micelle size distribution was characterized by image analysis using free, open-source ImageJ (1.54k).

#### 2.2.4. Nuclear Magnetic Resonance Spectroscopy (NMR)

Proton nuclear magnetic resonance (^1^H NMR) spectra were collected using a Bruker Ascend 500 MHz spectrometer (Bruker BioSpin GmbH, Rheinstetten, Germany) for the samples in CDCl_3_ as a solvent with TMS internal standard. Standard experimental conditions and the standard Bruker program were used.

#### 2.2.5. Differential Scanning Calorimetry

Differential scanning calorimetry (DSC) was conducted using a Mettler-Toledo DSC apparatus (Mettler-Toledo, Greifensee, Switzerland) equipped with a liquid nitrogen cooling system and an HSS8 ceramic sensor. Samples (approx. 5–10 mg) were placed in standard aluminium pans and subjected to a heating–cooling–heating cycle ranging from 0 to 300 °C at a constant heating rate of 10 °C/min under a nitrogen atmosphere.

#### 2.2.6. Hardness Measurements—Shore Method

Shore hardness was evaluated according to ISO 868 [68]. Three cylindrical samples (40 mm in diameter and 6 mm high) were made from each material. Hardness measurements were performed using a Bareiss HPE II-D digital durometer (Bareiss, Oberdischingen, Germany) at a controlled temperature of 21 ± 1 °C. Five independent indentations were made on each specimen with a dwell time of 5 s. The hardness values were recorded with an accuracy of 0.1 Shore D units, and the results were averaged for each material group.

#### 2.2.7. Hardness Measurements—Ball Indentation Method

Hardness measurements by the ball indentation method were performed using a Brinell hardness tester (EMCO-TEST Prüfmaschinen GmbH, Kuchl, Austria), in accordance with the PN-EN ISO 2039-14 standard [69]. A 5 mm diameter cemented carbide ball indenter was employed. The test pieces (40 mm in diameter and 6 mm in height) were subjected to test loads of 490.33 N and 306.46 N. Each measurement was performed in triplicate, with the load maintained for a dwell time of 30 s. The indentation hardness values were recorded with an accuracy of 0.01 MPa.

#### 2.2.8. Resistance to Bending Measurements

Bending tests were carried out using a Dynstat-type apparatus ((VEB Werkstoffprüfmaschinen Leipzig, Leipzig, Germany).) with a C-hammer, in accordance with the DIN 53435 (Testing of plastics, bending test and impact test on Dynstat test pieces) standards [70]. The dimensions of the test pieces were carefully maintained to meet the requirements of the Dynstat method (15 mm in length, 10 mm in width and 4 mm in height). The flexural strength was determined with an accuracy of 0.01 MPa.

#### 2.2.9. Tribological Tests

Tribological tests were performed using a TRN Tribometer operating in a ball-on-disc configuration (Anton Paar, Corcelles-Cormondrèche, Neuchâtel, Switzerland). The tribological tests were conducted in a reciprocating linear motion under dry contact conditions. The test parameters were selected based on preliminary studies. The tests were performed at a load of 10 N with a frequency of 5 Hz, over a friction distance of 100 m. The amplitude of motion was 10 mm. The maximum linear velocity was 15.71 cm/s. The tests were conducted at a temperature of 21 ± 1 °C and a humidity of 40 ± 5%. Balls with a diameter of 6 mm made of zirconium oxide (ZrO_2_) were used as a counter-sample. Following the tribological tests, the average surface area of the wear tracks was evaluated using a Surftest SJ-500 contact profilometer (Mitutoyo, Tokyo, Japan), with 8 measurements taken for each sample. Subsequently, volumetric wear was calculated using the formula:

(1) $$V_{w}=\frac{V}{F\cdot s}$$

where *V_w_*—volumetric wear [mm^3^/N·m], *V*—volume of material removed during friction [mm^3^], *V* = *P*∙*l*—where *P* is the average cross-sectional area of the wear scar, and *l* is the friction distance (10 mm), *F*—load [N], *S*—friction distance [m].

#### 2.2.10. Water Sorption Measurements

The material absorption method was carried out in accordance with PN-EN ISO 62:2008 [71]. The experiment involved specimens of neat PET-G and its composites containing zinc oxide (ZnO) at concentrations of 3%, 7%, and 10%, divided into two parallel series. In the first series, the samples were immersed in 20 mL of distilled water, while in the second series, they were placed in 20 mL of phosphate-buffered saline (PBS, pH 7.4). Prior to immersion, each specimen was accurately weighed. Both series were maintained under ambient conditions. After 7 days, the samples were removed, carefully dried to eliminate surface moisture, and reweighed to determine their final mass. The procedure was repeated three times for each material. The percentage of water absorption was calculated using the following formula:

(2) $$W_{w}=\frac{M_{n}-M_{s}}{M_{s}}\times 100[\%]$$

where *W_w_* is the ratio of water in the sample, *M_n_* is the mass of the swelled sample, and *M_s_* is the mass of the dry sample. The mass of each sample was measured to an accuracy of 0.0001 g.

#### 2.2.11. Assessment of Antimicrobial Activity

The antimicrobial properties of the studied composites were examined in vitro according to the previously described method [60,72,73]. The two standard strains of microorganisms were used: Gram-positive bacteria *Streptococcus oralis* ATCC 6249 (*S. oralis*) was purchased from the American Type Culture Collection (ATCC, Manassas, VA, USA) and the yeast-type fungus *Candida albicans* ATCC 10231 (*C. albicans*) was obtained from the American Type Culture Collection (ATCC, Manassas, VA, USA). Sterilized square specimens of the studied composites were introduced individually into 2 mL of bacterial or fungal suspensions in tryptone water, which contained 1.5 × 10^5^ CFU/mL (CFU, colony-forming units) of *S. oralis* or *C. albicans*. Suspensions of bacteria or fungi in tryptone water were tested as a positive control. As a negative control, pure tryptone water was tested. All of the samples with microorganism suspensions were incubated in a shaking incubator for 17 h at 35 °C for *C. albicans* and at 37 °C for *S. oralis*. After incubation, 20 µL of each suspension was seeded onto Columbia agar with 5% sheep blood plates for *S. oralis*, and Sabouraud agar plates for *C. albicans*. The Columbia agar and Sabouraud agar were purchased from bioMérieux (Marcy l’Etoille, France). The cultured plates were incubated at 35 °C for 48 h (yeast) or 37 °C for 24 h (bacteria). Then, the number of bacterial or fungal colonies was counted with a colony counter (ProtoCOL 3 PLUS, Synbiosis, Frederick, MD, USA). The materials’ antibacterial (ABE) or antifungal (AFE) efficacy was calculated according to the following equation:

(3) $$\frac{V_{c}-V_{t}}{V_{c}}\times 100[\%]$$

where V_c_ was the number of microorganism colonies from the positive control (blank), and V_t_ was the number of microorganism colonies of the test specimen.

## 3. Results and Discussion

In the present study, a composite based on a copolymer of ethylene terephthalate and 1,4-cyclohexanedimethanol terephthalate (PET-G) was prepared with the addition of zinc oxide (ZnO) at three different concentrations: 3%, 7%, and 10% by weight. Since achieving uniform dispersion of ZnO within the polymer matrix poses a significant challenge due to the high viscosity of the molten polymer, which hinders homogeneous mixing, a two-stage fabrication process employing a solvent-based approach was utilized.

In the first stage, PET-G was dissolved in dichloromethane (DCM) to obtain a uniform, clear solution. Subsequently, appropriate amounts of ZnO were added to the dissolved polymer at concentrations of 3%, 7%, and 10% by weight and mixed until a homogeneous structure was achieved. Following thorough dispersion of ZnO within the solution, the mixture was cast into silicone molds and dried to allow solvent evaporation, yielding solid yet flexible composite films.

**Figure 1:**
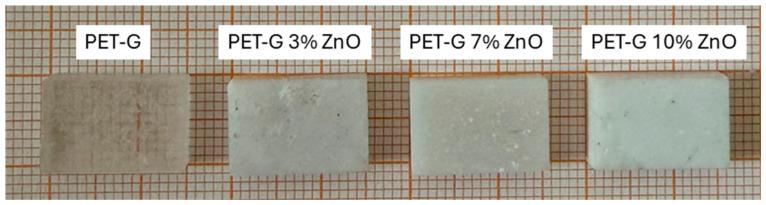
Samples prepared from PET-G polymer and its composites with ZnO.

After complete solvent evaporation, these intermediate samples were melted at 260 °C and thermoformed to produce the final casts. This approach resulted in the production of highly homogeneous composite materials (Figure 1), characterized by ZnO particles evenly dispersed throughout the PET-G matrix, which is crucial for the properties, functional performance, and reliability of the final splints.

**Figure 2:**
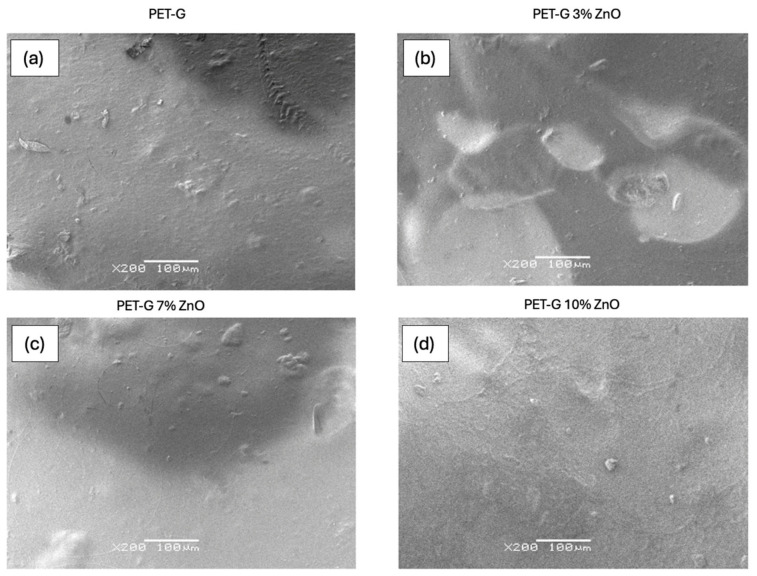
Representative SEM-BSE images at 200× magnification of a pure polymer PET-G surface (**a**) and a surface composition PET-G with added zinc oxide (ZnO) (**b**–**d**).

The SEM micrographs presented in Figure 2 (magnification ×200, scale bar 100 μm) illustrate the surface morphology of PET-G and its composites modified with zinc oxide (ZnO). All samples exhibited a continuous polymer matrix without visible cracks, delamination, or macroscopic defects indicative of material degradation, suggesting the effective incorporation of the inorganic filler into the polymer phase. The unmodified PET-G sample displayed a relatively uniform and smooth surface, with only minor local irregularities and isolated traces associated with the processing procedure.

With increasing zinc oxide (ZnO) content, a slight, gradual increase in surface heterogeneity was observed, accompanied by the appearance of small protrusions and regions of altered topography. These features can be attributed to the presence of ZnO particles or small agglomerates dispersed within the polymer matrix. The composites containing lower ZnO concentrations exhibited only subtle changes in surface morphology compared with neat PET-G, whereas samples with higher ZnO content showed a greater number of surface irregularities and more pronounced topographical variations. Nevertheless, these differences remained relatively minor and did not indicate any deterioration of the composite structure. Despite the local occurrence of inorganic phase clusters, no clear evidence of phase separation or large agglomerate formation, which would indicate ineffective homogenization, was observed. The distribution of ZnO appeared relatively uniform, and the observed microstructural changes confirmed the successful modification of PET-G with zinc oxide.

The resulting microstructure indicates that the structural integrity and homogeneity of the material were preserved while simultaneously introducing a functional phase potentially responsible for the bioactive and antimicrobial properties of the composites. Similar microstructural features associated with the incorporation of zinc oxide into polymeric materials have been reported in the scientific literature [63,64], where detailed analyses confirmed the effective dispersion of ZnO within various polymer matrices, providing a valuable reference for comparison with the results obtained in the present study.

In a subsequent stage of the investigation, differential scanning calorimetry (DSC) was performed to characterize the thermal behaviour, structural stability, and phase transitions of the prepared composites. Analysis of the neat polymer sample revealed a glass transition event with a Tg of 80 °C, followed by an endothermic peak corresponding to the melting process at Tm = 255 °C (Figure S5). It is worth noting that the melting signal was relatively weak, which is associated with the reduced crystallinity of PET-G.

In the investigated composite samples, the addition of ZnO did not significantly affect the glass transition of the polymer matrix. Interestingly, the melting endothermic peak was no longer observed in these formulations, suggesting structural reorganization within the material. No substantial differences were observed among the individual ZnO-containing composites, regardless of the filler concentration. The thermal response of all modified materials remained comparable, indicating that even the lowest ZnO addition was sufficient to induce similar changes in the thermal behaviour of the PET-G matrix. This suggests that the inorganic additive acts as a plasticizer, hindering crystallization. Similar phenomena have already been reported in the literature for polymer composites containing inorganic fillers [74,75,76]. These observations regarding the thermal properties of the composites are of great importance, as they directly influence the processing parameters and the feasibility of manufacturing new clinical devices.

Further analyses focused on the mechanical properties of the resulting composites, beginning with an evaluation of how the addition of ZnO affects the material’s resistance to indentation. Initially, the Shore D method was employed to measure the samples, as it is designed for plastics with considerable hardness. These measurements were performed in accordance with the ISO 868 standard [68], and the results are presented in Figure S6.

It can be observed that the addition of ZnO to the PET-G polymer at concentrations of 3% and 7% leads to a reduction in the material’s hardness. However, at the highest concentration of 10%, a slight increase is observed compared to neat PET-G and the composites containing lower amounts of the additive. Among the modified materials, the 3% and 7% ZnO composites exhibited very similar hardness values, indicating that increasing the filler content within this range had only a limited influence on indentation resistance. In contrast, the composite containing 10% ZnO showed a slight recovery in hardness, suggesting that a higher filler loading may partially compensate for the initial softening effect observed at lower ZnO concentrations. Nevertheless, these differences are minor and unlikely to affect the clinical performance of the material. Similar trends have been observed in other scientific studies, demonstrating that the addition of ZnO to polymers often causes an initial decrease in hardness, which then stabilizes or even slightly increases at higher filler loadings. This behaviour is typically attributed to changes in the crystal structure and specific particle–matrix interactions [77,78]. Such findings confirm that the influence of ZnO on hardness is complex, as it is governed by both the concentration of the filler and its synergy with the base polymer matrix.

In order to perform more accurate measurements, hardness was further analysed using the ball indentation method in accordance with the PN-EN ISO 2039-1 standard [69]. As shown in Figure S7, the addition of ZnO had a minimal effect on the overall hardness of the PET-G material. Specifically, across all tested concentrations, a consistent slight reduction in hardness of approximately 10 MPa was observed. The hardness values obtained for the individual ZnO-containing composites were very similar, with no clear concentration-dependent trend observed among the modified materials. This indicates that increasing the ZnO content from 3% to 10% did not produce any measurable differences in indentation hardness beyond the slight reduction observed for all composites relative to neat PET-G. It is worth noting that none of the ZnO-modified blends exceeded the hardness of the base material. This indicates that the filler does not cause excessive brittleness or hardening. This minor decline in mechanical performance is likely correlated with the reduction in the degree of crystallinity, as indicated by the DSC analysis.

Overall, the addition of ZnO did not result in significant differences in hardness between the tested ZnO-modified PET-G materials and the base material. These findings are consistent with previous studies [79], which analysed the influence of various additives on polymer hardness and reported similarly minor or negligible effects on mechanical performance. Considering the potential application of the developed materials for the fabrication of dental splints, further investigation focused on properties critical for clinical use, such as flexural strength (which reflects the forces encountered during biting) and abrasion resistance.

Bending tests of PET-G and its composites were performed in accordance with the DIN 53435:2018-09 [70] using a Dynstat-type apparatus. The procedure involved gradually increasing the load until the sample underwent deformation. During testing, it was observed that none of the samples exhibited brittle fracture. Instead, they showed significant bending accompanied by elongation. This absence of rupture (Figure S8b) indicates that the flexural strength limit of the materials was not exceeded under the applied conditions. However, a decrease in flexural resistance was observed following the addition of ZnO to the polymer. Based on Figure S8a, it can be concluded that the incorporation of ZnO reduced flexural resistance by approximately 10–15 MPa. The differences in flexural resistance among the individual ZnO-containing composites were relatively small, indicating that increasing the ZnO content did not result in a pronounced concentration-dependent effect. All modified materials exhibited a comparable reduction in bending resistance relative to neat PET-G while maintaining their ductile behaviour during testing. This effect is likely attributable to the inherent inhomogeneity of the composite system resulting from the nature of its components. ZnO, as an inorganic substance, does not mix homogeneously with the organic PET-G polymer but is instead dispersed throughout the material structure. Such inhomogeneity can lead to local concentrations of inorganic particles, which may weaken the material structure and affect its mechanical properties. Although the PET-G/ZnO composites remained ductile and did not fracture, they exhibited lower bending resistance than neat PET-G. Similar findings regarding the influence of inorganic fillers on the bending behaviour of polymers have previously been reported in the literature [79], confirming that system inhomogeneity often leads to a reduction in certain mechanical properties, including bending resistance.

The wear track measurements obtained using a profilometer for PET-G-based polymers containing 3%, 7%, and 10% ZnO are presented in Figure S9. The 10% ZnO composite showed the lowest volumetric wear (highest wear resistance), while the 3% ZnO variant exhibited the most significant wear, exceeding that of neat PET-G.

Additionally, a clear correlation was observed between the material’s surface hardness (Shore D, Figure S6) and its wear resistance. This distinct concentration-dependent wear behavior can be explained by a transition in the filler’s role from a micro-stress concentrator to a load-bearing network. At lower loadings (3 wt.%), widely dispersed ZnO particles act as microscopic defects that facilitate micro-ploughing and suffer from particle pull-out, with the dislodged particles acting as third-body abrasives that accelerate wear. Conversely, at 10 wt.%, the reduced inter-particle distance allows the densely packed ZnO particles to form an interconnected, supportive framework. This network effectively anchors the particles within the matrix, prevents pull-out, and transfers the applied load from the polymer chains to the rigid inorganic filler. Furthermore, the high concentration of ZnO at the sliding interface promotes the formation of a stable, compacted mechanically mixed layer (MML) composed of comminuted ZnO and polymer debris, which acts as a protective shield suppressing abrasive wear.

These observations are consistent with literature findings [80], where increasing the content of inorganic additives, such as ZnO, leads to improved hardness and tribological behaviour of polymeric materials, which is crucial for their intended clinical applications.

Taking into account the target environment of the designed materials, further research was conducted to determine how the addition of ZnO affects the material’s water absorption. This property is of particular importance since dental inserts operate in the oral cavity, where they remain in constant contact with saliva and other aqueous media. The water absorption of PET-G and its ZnO-containing composites was measured in accordance with PN-EN ISO 62:2008 [71]. Samples of the materials were placed either in deionized water or in phosphate-buffered saline (PBS, pH 7.4) and left for 7 days to reach fluid sorption equilibrium.

Notably, the differences in fluid sorption between water and the buffer were minimal, remaining within a 1% range. Therefore, such low values should not cause significant changes in the physicochemical or mechanical properties of the materials during clinical use. The results shown in Figure S10 indicate that increasing the ZnO concentration in the material resulted in slightly higher sorption of PBS. Among the ZnO-containing composites, the increase in PBS sorption was gradual, with the highest values recorded for the material containing 10% ZnO. Nevertheless, the differences between the individual composites remained small, indicating that the filler concentration had only a limited influence on the fluid uptake behaviour of the modified materials. In contrast, for deionized water, no significant differences were observed between the ZnO-modified samples and neat PET-G. Furthermore, the base material exhibited lower sorption in PBS than in deionized water.

This behaviour can be attributed to the relatively low polarity of the PET-G polymer compared to the aqueous environments. Similar observations have been reported previously [81,82], where low-polarity materials demonstrated reduced sorption in highly polar media, which explains the lower absorption values observed for buffers compared to deionized water.

To ensure a clear and systematic presentation of the obtained results, as well as to facilitate their comparison and interpretation, the key parameters characterizing the investigated materials are summarized in Table 1. The presented data comprise the results of analyses performed on unmodified PET-G and ZnO-containing composites with different filler concentrations, enabling a comprehensive assessment of the influence of zinc oxide incorporation on the properties of the investigated materials.

**Figure 3:**
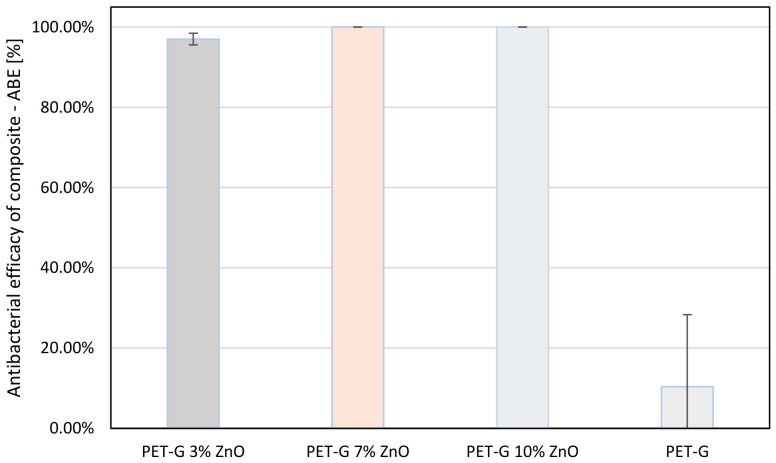
Mean antibacterial efficacy against *Streptococcus oralis* ATCC 6249.

The key aspect of the research on the proposed materials was to determine whether the addition of ZnO to the polymer matrix would affect (or rather improve) its antimicrobial properties. To this end, tests were conducted using two model microorganisms that are common commensal components of the oral cavity microbiota. While typically harmless, these organisms can act as opportunistic pathogens and cause infections under specific medical conditions [83]. Since both bacteria and fungi can contribute to such complications, the antimicrobial potential of the composites was evaluated against representative species: *Streptococcus oralis* (bacterium) and *Candida albicans* (fungus). The results are presented in Figure 3.

**Figure 4:**
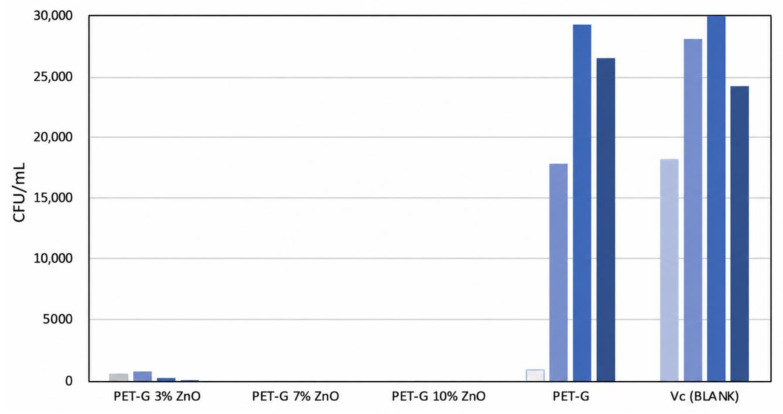
Number of CFU/mL after 17 h of incubation with the composite (Vt). For each tested material, four bars (shown in different shades of blue)represent four independent experimental replicates performed under identical conditions. Vc (BLANK) corresponds to the control sample without the composite material.

As shown in Figure 3, the incorporation of ZnO into the polymer matrix significantly enhanced the material’s antibacterial activity. A clear concentration-dependent improvement in antibacterial efficacy was observed between the composites containing 3% and 7% ZnO. While the 3% ZnO composite already exhibited very high antibacterial activity (approximately 97% inhibition), complete inhibition of *S. oralis* growth was achieved only for the composites containing 7% and 10% ZnO. No further improvement was observed when the ZnO concentration increased from 7% to 10%, suggesting that 7% ZnO was sufficient to reach the maximum antibacterial effect under the applied experimental conditions. Notably, ZnO concentrations of 7% and 10% resulted in complete inhibition of *S. oralis* colony growth within the tested system, as illustrated in Figure 4.

While ZnO is widely recognized for its potent antibacterial properties [84], its impact on the overall characteristics of the composite must be carefully balanced. Therefore, it is paramount to determine which concentration provides sufficient antimicrobial efficacy without compromising the material’s integrity, based on the findings of this pilot study. In contrast, the results regarding antifungal activity remain inconclusive. This is consistent with the fact that ZnO typically does not exhibit strong antifungal properties [85]. Although increasing the ZnO concentration appeared to slightly enhance the cytotoxic effect against *C. albicans*, the values obtained in this preliminary phase are not substantial enough to be considered conclusive and therefore require further investigation.

## 4. Conclusions

This work proposes a method for preparing a novel dental composite based on a PET-G copolymer modified with ZnO. Thermal analysis confirmed that the inorganic additive does not impair the material’s processability or the feasibility of fabricating devices for various clinical applications. Furthermore, mechanical testing demonstrated that, even at the highest ZnO loading, no significant deterioration in the material’s structural integrity was observed. Key findings of this study include the effect of the inorganic oxide additive on water sorption and the sorption of physiological fluid models, as well as the successful demonstration of the composite’s antibacterial efficacy. These results are of great importance for the development of future dental products and fill a research gap that has not previously been explored in the context of various types of dental splints. Within the limitations of this preliminary in vitro study of antimicrobial efficacy, it was shown that filler concentrations between 3% and 7% should be considered the favourable range for achieving a balance between material stability and antibacterial activity.

Further studies with a greater number of replicates are necessary to determine the precise ZnO concentrations required to ensure both satisfactory antibacterial and antifungal properties. Future research should also incorporate a broader range of model microorganisms to more accurately reflect the complexity of the human oral microbiota. In conclusion, our findings underscore the potential of ZnO-modified polymers to redefine current standards in the fabrication of functional, bioactive dental inserts. The outcomes of our study may also pave the way for a new generation of dental splints that not only provide mechanical support but also actively contribute to maintaining oral health and preventing opportunistic infections.

## Figures and Tables

**Table 1:** Summary of the test results obtained for neat PET-G and PET-G/ZnO composites. Values are presented as mean ± standard deviation (SD).

		PET-G	PET-G 3% ZnO	PET-G 7% ZnO	PET-G 10% ZnO
Differential Scanning calorimetry (°C)	T_g_	80	80	80	80
	T_m_	255	-	-	-
Hardness measurements Shore D method (° Shore D)		74 ± 1.2	71 ± 3.8	71 ± 1.5	76 ± 2.5
Hardness measurements ball indentation method (MPa)		109.98 ± 14.72	85.07 ± 13.02	105.57 ± 33.40	99.97 ± 12.60
Resistance to bending measurements (MPa)		78.16 ± 4.68	54.08 ± 6.89	69.70 ± 1.33	59.19 ± 2.25
Tribological tests (mm^3^/(N⋅m))		0.00154 ± 0.00094	0.00269 ± 0.00125	0.00199 ± 0.00097	0.00149 ± 0.00088
Water sorption measurements (%)	DI water	0.90 ± 0.25	0.96 ± 0.55	0.94 ± 0.15	0.89 ± 0.16
	PBS buffer pH 7.4	0.66 ± 0.12	0.70 ± 0.33	1.04 ±0.46	1.23 ± 0.07

## Data Availability

The original contributions presented in this study are included in the article/Supplementary Material.

## Supplementary Materials

The following supporting information can be downloaded at https://www.mdpi.com/article/10.3390/ma19143033/s1, Figure S1: NMR spectrum of commercial PET-G in CDCl_3_; Figure S2: SEM micrographs of pristine ZnO powder (98% purity, Warchem^®^) obtained at magnifications of ×2000 (A) and ×5000 (B), showing the morphology and aggregation of ZnO particles; Figure S3: TEM micrographs of pristine ZnO powder (98% purity, Warchem^®^); Figure S4: Size distribution of the raw ZnO particles obtained via quantitative TEM analysis; Figure S5: DSC analysis of PET-G and its composites with ZnO; Figure S6: Results of hardness tests performed using the Shore D method; Figure S7: Ball indentation hardness measurement results for PET-G and its composites with ZnO; Figure S8: Results of bending resistance measurements (A) and photograph of the sample after testing (B); Figure S9: Results of profilometric measurements of wear marks on PET-G and its composites containing ZnO; Figure S10: Water and PBS buffer sorption assay results after 7 days; Table S1: Summary of statistical parameters for the grain size distribution of the pristine ZnO powder calculated from TEM analysis.
