# Supplementary material for: Antibacterial PET-G/ZnO Composites: A New Approach to Relaxation Splint Materials in the Treatment of Bruxism
Source: Materials (Basel). 2026 Jul 14;19(14):3033. doi: 10.3390/ma19143033 (PMC13413869; doi:10.3390/ma19143033)
Supplement: Supplementary file 1 [file materials-19-03033-s001.zip › materials-4385203-supplementary.pdf]

Antibacterial PET-G/ZnO Composites: A New Approach to Relaxation Splint Materials in the Treatment of Bruxism

Sandra Wąsik [1,2], Rafał Bielas [3], Roksana Wygoda [2], Mateusz Wojciechowski [4], Magdalena Tarnacka [5], Krzysztof Aniołek [2], Anna Mertas [6], Maciej Zubko [2], Karsten Manterys [2], Izabela Barszczewska-Rybarek [7], Stefan Baron [4] and Małgorzata Karolus [2,*]

[1] International Environmental Doctoral School, Faculty of Natural Sciences, University of Silesia, Bedzinska 60, 41-200 Sosnowiec, Poland; sandra.wasik@us.edu.pl
[2] Institute of Materials Engineering, University of Silesia in Katowice, 75 Pulku Piechoty 1A, 41-500 Chorzow, Poland; roksana.wygoda@us.edu.pl (R.W.); krzysztof.aniolek@us.edu.pl (K.A.); maciej.zubko@us.edu.pl (M.Z.); karsten.manterys@us.edu.pl (K.M.)
[3] Department of Pharmacognosy and Phytochemistry, Faculty of Pharmaceutical Sciences in Sosnowiec, Medical University of Silesia in Katowice, Jagiellonska 4, 41-200 Sosnowiec, Poland; rafal.bielas@sum.edu.pl
[4] Department of Temporomandibular Disorders, Medical University of Silesia in Katowice, Traugutta Sq. 2, 41-800 Zabrze, Poland; m.wojciechowski@sum.edu.pl (M.W.); sbaron@sum.edu.pl (S.B.)
[5] Institute of Physics, Faculty of Science and Technology, University of Silesia in Katowice, 75 Pulku Piechoty 1A, 41-500 Chorzow, Poland; magdalena.tarnacka@us.edu.pl
[6] Department of Microbiology and Immunology, Faculty of Medical Sciences in Zabrze, Medical University of Silesia, Jordana 19 Street, 41-808 Zabrze, Poland; amertas@sum.edu.pl
[7] Department of Physical Chemistry and Technology of Polymers, Faculty of Chemistry, Silesian University of Technology, 44-100 Gliwice, Poland; izabela.barszczewska-rybarek@polsl.pl
* Correspondence: malgorzata.karolus@us.edu.pl

LIST OF SUPPLEMENTARY FIGURES

FIGURE S1. NMR SPECTRUM OF COMMERCIAL PET-G IN $CDCL_3$. 3
FIGURE S2. SEM MICROGRAPHS OF PRISTINE ZNO POWDER (98% PURITY, WARCHEM®) OBTAINED AT MAGNIFICATIONS OF ×2000 (A) AND ×5000 (B), SHOWING THE MORPHOLOGY AND AGGREGATION OF ZNO PARTICLES....................3
FIGURE S3. TEM MICROGRAPHS OF PRISTINE ZNO POWDER (98% PURITY, WARCHEM®). 4
FIGURE S4. SIZE DISTRIBUTION OF THE RAW ZNO PARTICLES OBTAINED VIA QUANTITATIVE TEM ANALYSIS. 4
FIGURE S5. DSC ANALYSIS OF PET-G AND ITS COMPOSITES WITH ZNO. 5
FIGURE S6.RESULTS OF HARDNESS TESTS PERFORMED WITH SHORE D METHOD. 6
FIGURE S7. BALL INDENTATION HARDNESS MEASUREMENT RESULTS FOR PET-G AND ITS COMPOSITES WITH ZNO. 6
FIGURE S8.RESULTS OF RESISTANCE TO BENDING MEASUREMENTS (A) AND SAMPLE AFTER TESTS (B). .7
FIGURE S9.RESULTS OF PROFILOMETRIC MEASUREMENTS OF WEAR MARKS ON PET-G AND ITS COMPOSITES CON-TAINING ZNO. 7
FIGURE S10. WATER AND PBS BUFFER SORPTION ASSAY RESULTS AFTER 7 DAYS. 8

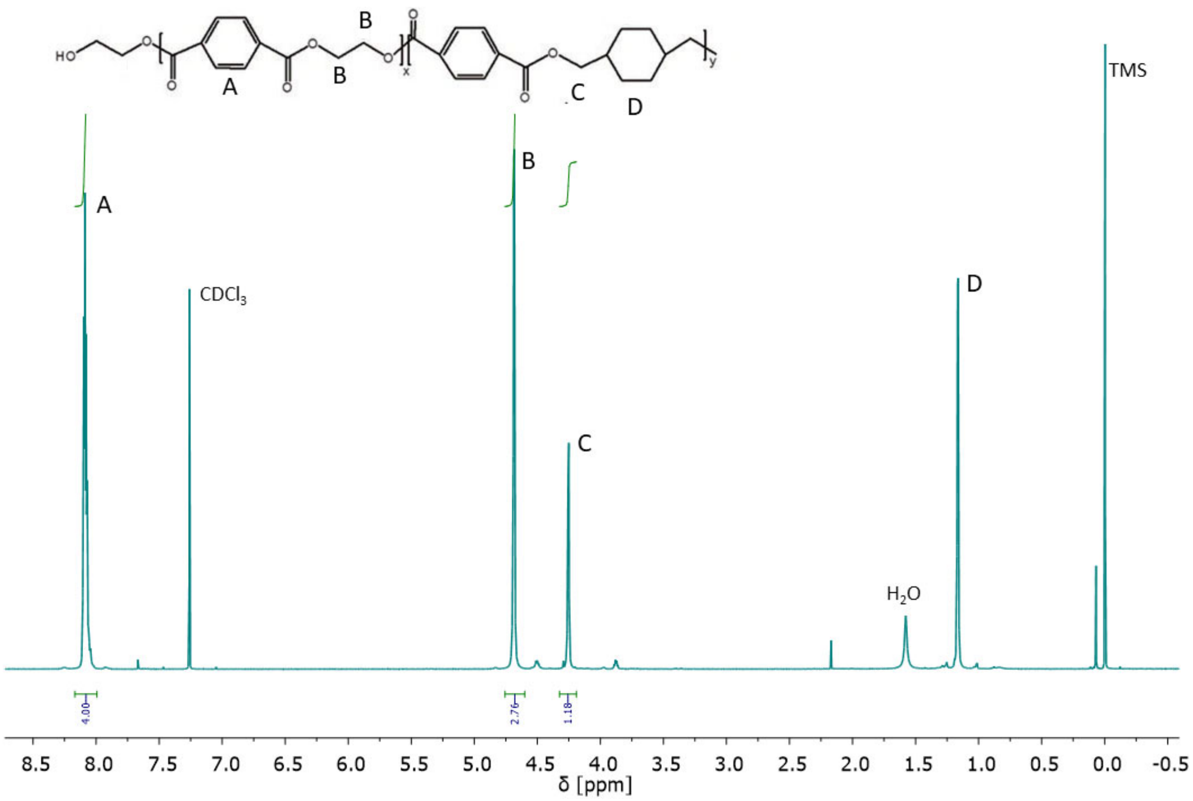

Figure S1. NMR spectrum of commercial PET-G in $CDCl_3$.

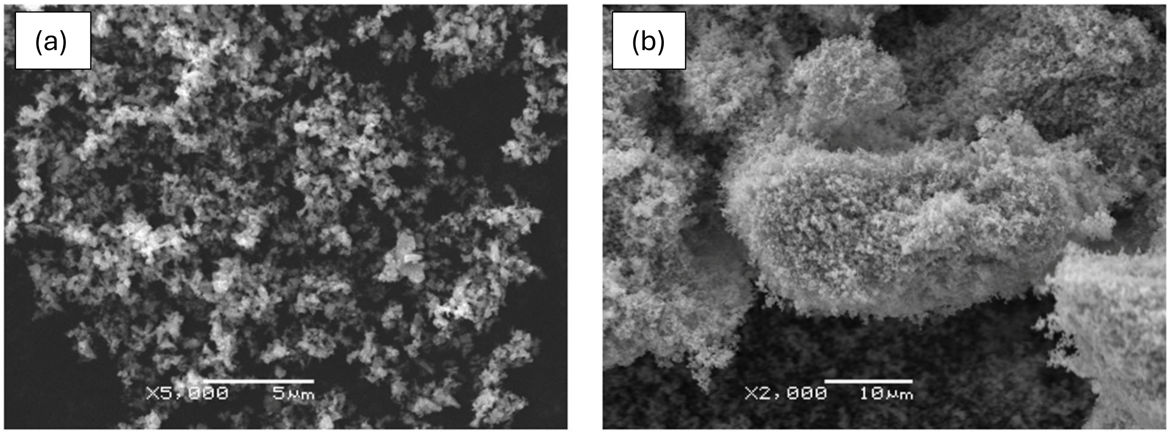

Figure S2. SEM micrographs of pristine ZnO powder (98% purity, Warchem®) obtained at magnifications of ×2000 (a) and ×5000 (b), showing the morphology and aggregation of ZnO particles.

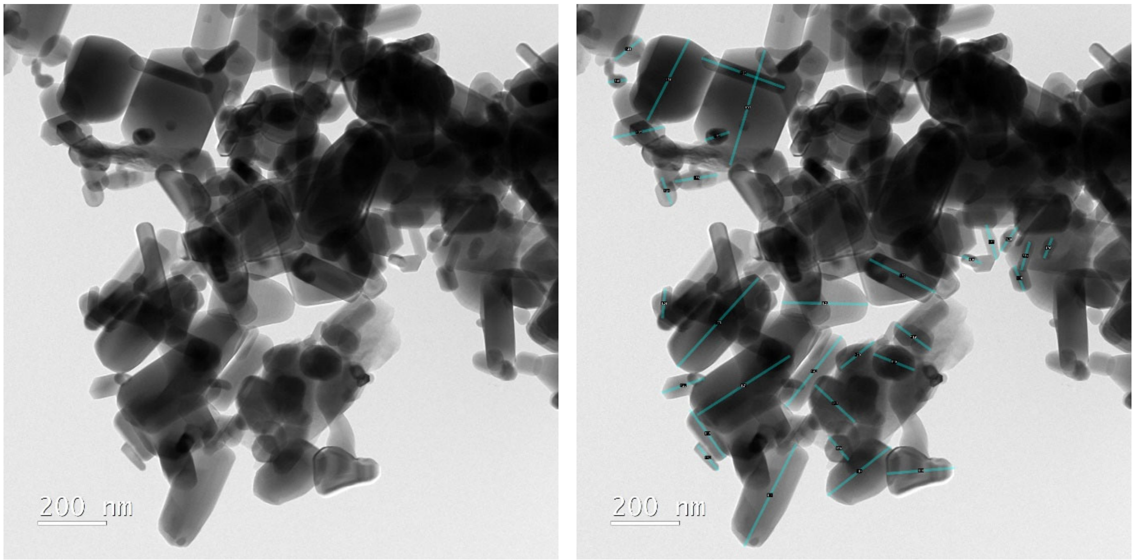

Figure S3. TEM micrographs of pristine ZnO powder (98% purity, Warchem®).

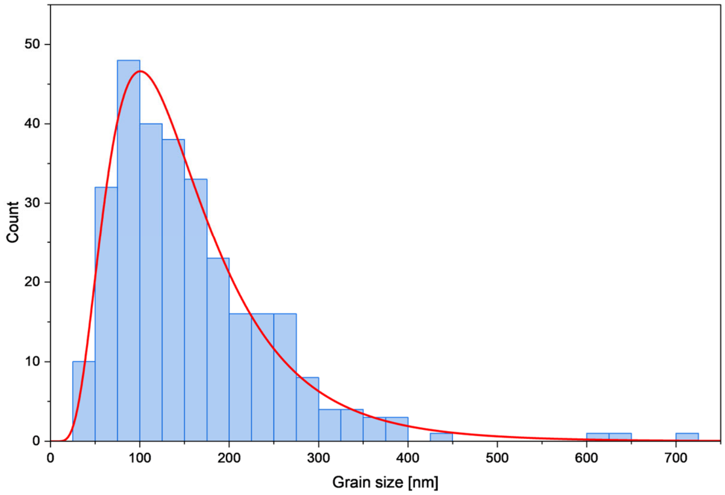

Figure S4. Size distribution of the raw ZnO particles obtained via quantitative TEM analysis.

Bright-field TEM observations of the ZnO_2 powder revealed a nanocrystalline morphology with a pronounced tendency toward agglomeration. The material consists mainly of faceted particles with polygonal, plate-like and short-prismatic shapes. A fraction of nearly equiaxed grains is also visible, while some particles show elongated morphologies. The observed grains are mostly well defined and display relatively sharp external boundaries, suggesting the formation of discrete crystalline particles. In several areas, small particles are attached to or located on the surfaces of larger grains.

The grain size distribution is broad and clearly asymmetric. Most of the measured particles are in the nanoscale range, approximately 50-250 nm, with the highest population

centred at 90–120 nm. The histogram shows a right-skewed distribution with a long tail toward larger sizes. A limited number of coarse particles or aggregates with apparent sizes above 300 nm is also present, including isolated objects approaching 600–700 nm. This indicates that the ZnO powder is not monodisperse, but consists of a dominant population of fine nanograins accompanied by a smaller fraction of larger particles or agglomerates. The fitted distribution matches the experimental histogram reasonably well in the main particle-size range, especially near the distribution maximum. Deviations in the high-size region are most likely associated with the limited number of large particles and with the difficulty of distinguishing individual grains in strongly overlapping agglomerates.

Table S1. Summary of statistical parameters for the grain size distribution of the pristine ZnO powder calculated from TEM analysis.

N total	Mean [nm]	Std. Dev. [nm]	Mode [nm]	Minimum [nm]	Median [nm]	Maximum [nm]	Range [nm]
298	157,3	93,2	57,5	34,0	136,8	717,8	683,9

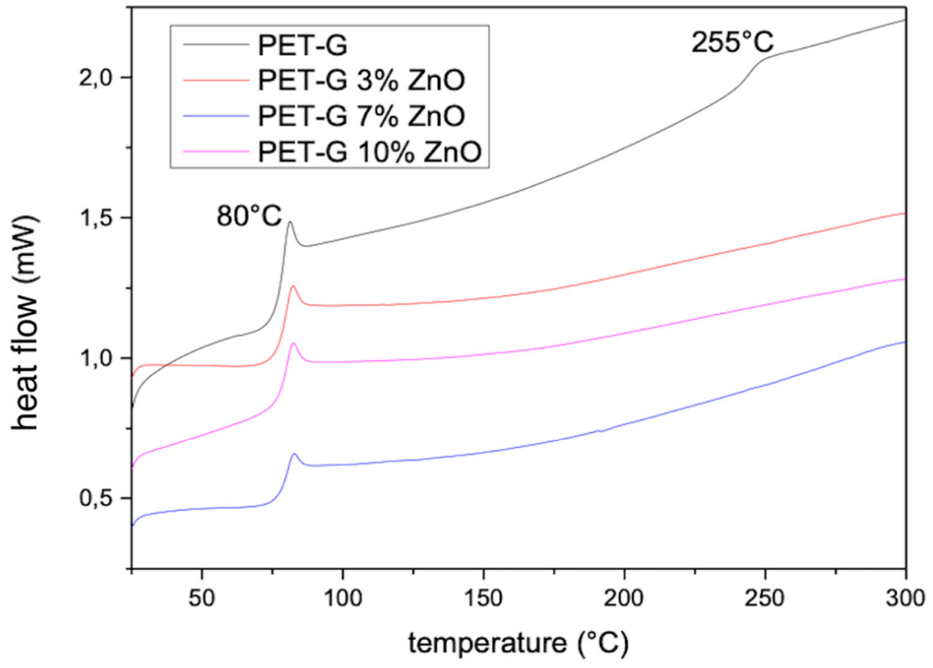

Figure S5. DSC analysis of PET-G and its composites with ZnO.

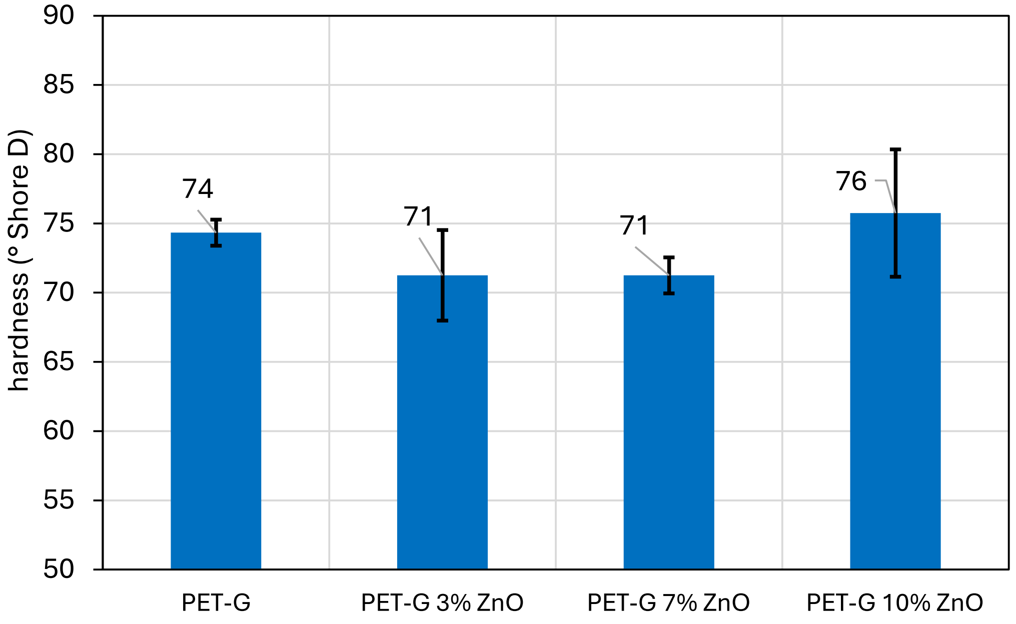

Figure S6. Results of hardness tests performed with Shore D method.

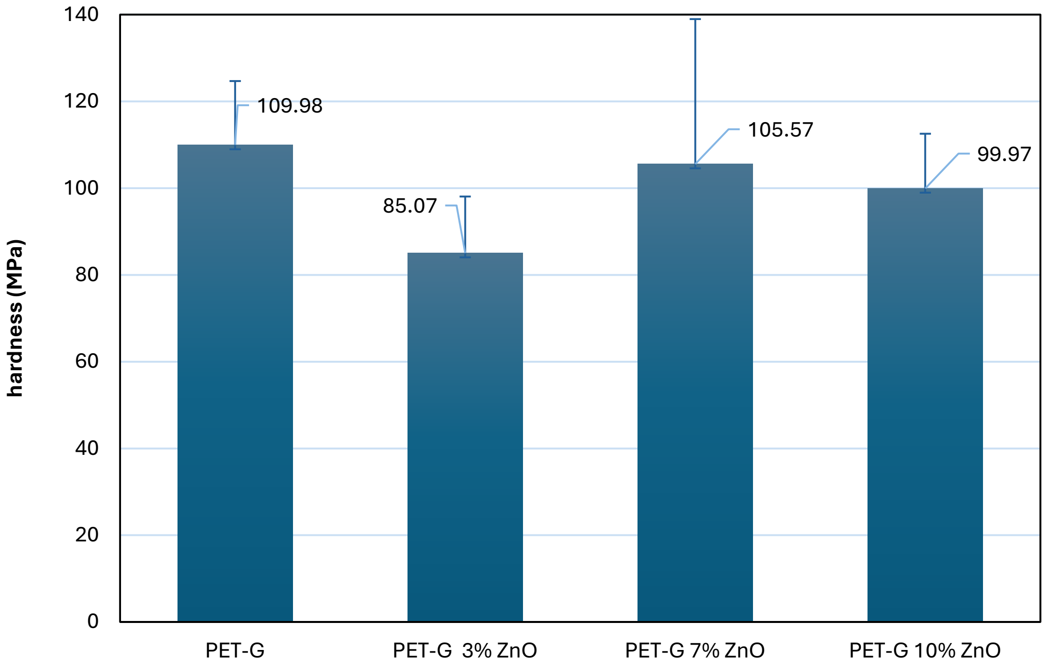

Figure S7. Ball indentation hardness measurement results for PET-G and its composites with ZnO.

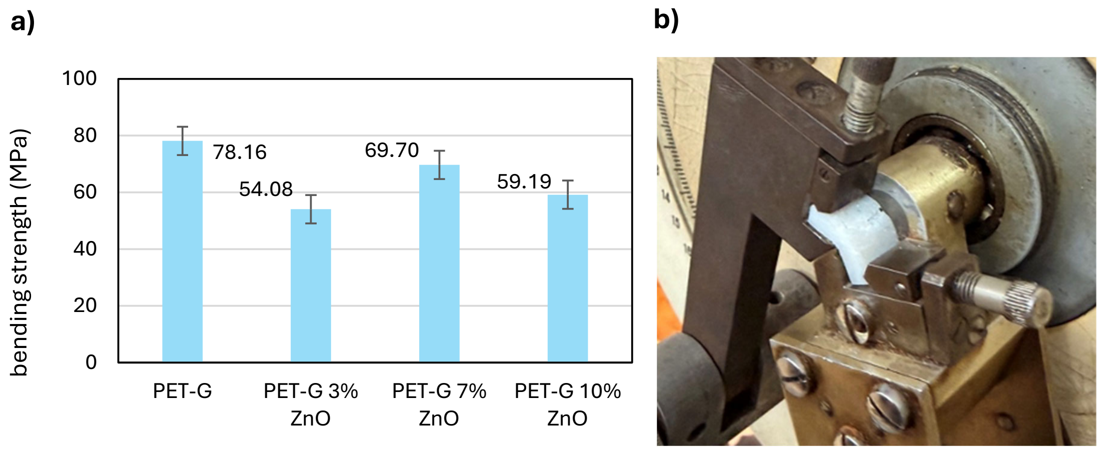

Figure S8. Results of resistance to bending measurements (a) and sample after tests (b).

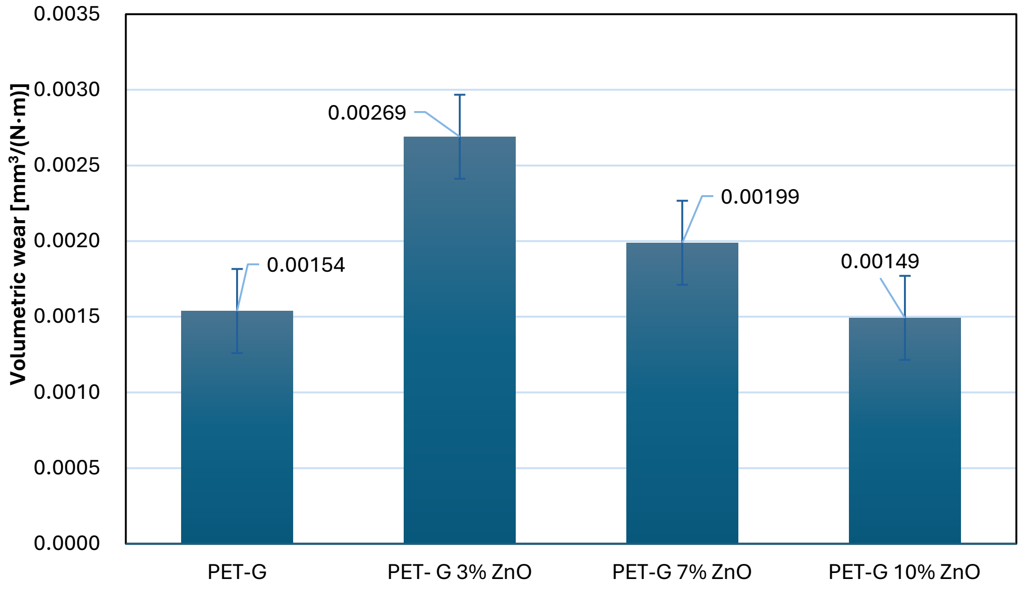

Figure S9. Results of profilometric measurements of wear marks on PET-G and its composites con-taining ZnO.

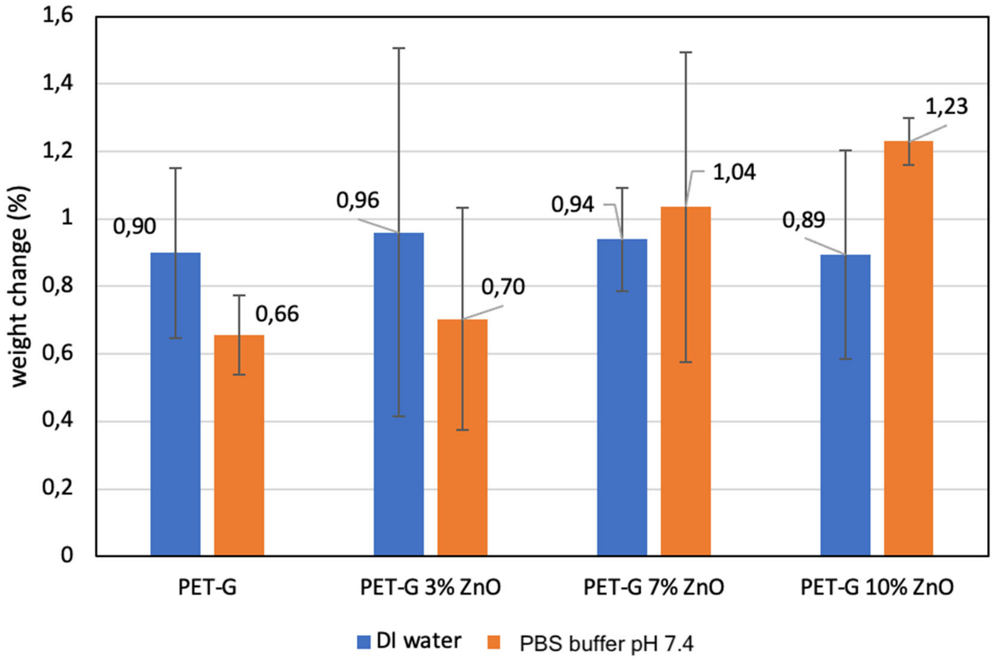

Figure S10. Water and PBS buffer sorption assay results after 7 days.
